# Fractional 1064 nm Nd:YAG picosecond laser for Asian skin rejuvenation: clinical efficacy and the role of photoprotective behaviours

**DOI:** 10.1007/s10103-025-04453-4

**Published:** 2025-04-23

**Authors:** Thai Van Thanh Le, Phuong Thao Nguyen, Vi Anh Le, Quoc Hung Ta, Alessandra Zevini, Daniela Martinelli, Riccardo Barini

**Affiliations:** 1 Department of Dermatology and Skin Aesthetics, University Medical Center at Ho Chi Minh City, Ho Chi Minh City, Vietnam; 2 Department of Dermatology and Venereology, Faculty of Medicine, University of Medicine and Pharmacy at Ho Chi Minh City, Ho Chi Minh City, Vietnam; 3 El. En. Group, Calenzano, Italy

**Keywords:** Picosecond Nd:YAG 1064 Nm laser, Skin rejuvenation, Fractional laser therapy, Photoprotective behaviors, Asian skin

## Abstract

Skin rejuvenation is a vital aspect of dermatology aimed at countering intrinsic and extrinsic aging effects, such as wrinkles, dyspigmentation, and texture irregularities. This study evaluates the efficacy and safety of the fractional 1064 nm Nd:YAG picosecond laser in a Vietnamese cohort, addressing unique challenges posed by Asian skin’s high melanin content. 44 Vietnamese participants treated at the University Medical Center of Ho Chi Minh City for facial skin rejuvenation were retrospectively analyzed. Outcomes were assessed via standardized imaging and patient-reported satisfaction scores, with statistical analyses applied to evaluate changes in skin indices and identify epidemiological correlations. Measurements using the VISIA^®^ system indicated statistically significant reductions in wrinkles, spots, and texture irregularities starting from the first month of treatment (*p* < 0.01) and sustained through three months (*p* < 0.0001). Adverse effects were minimal, with transient erythema being the most common (68.18%), and pain levels were generally mild to moderate. Epidemiological analysis revealed that prolonged sun exposure adversely affected UV spot improvement (*p =*  0.01), while consistent mask-wearing correlated with enhanced outcomes for pigmentation reduction (*p =*  0.03). This study underscores the fractional 1064 nm Nd:YAG picosecond laser’s effectiveness as a safe and versatile tool for skin rejuvenation, particularly for Asian populations prone to post-inflammatory hyperpigmentation. Recommendations for consistent photoprotection and addressing environmental factors further optimize results. Future studies should explore long-term outcomes and comparative efficacy with alternative modalities.

## Clinical trial number

Not applicable.

## Introduction

Skin rejuvenation is a critical area of dermatology, aiming to counteract the effects of intrinsic and extrinsic aging processes. Aging and photoaging contribute to a range of cutaneous changes, including wrinkles, loss of elasticity, and pigmentation irregularities, which collectively compromise skin quality and appearance [1]. These changes stem from structural and compositional alterations in the dermis, notably collagen degradation and the diminished functionality of elastin fibers, often exacerbated by environmental factors like ultraviolet (UV) radiation [2].

Facial rejuvenation is a field that has seen significant advancements in recent years, catering to the growing demand for non-invasive and minimally invasive procedures. Surgical interventions remain a cornerstone for comprehensive rejuvenation, offering long-lasting results through facelifts and other contouring procedures. Botulinum toxin and filler injections have revolutionized the approach to anti-aging, providing quick and effective solutions to wrinkles and volume loss with minimal downtime. However, such invasive approaches carry the potential for adverse events, including infection, scarring, and anesthesia complications, therefore they are not usually offered as first-choice procedures [3, 4]. Consequently, there has been an increased focus on non-invasive approaches that provide significant improvement with minimal risks and recovery time.

Lasers can address a range of skin concerns, including dyspigmentation, texture irregularities, and fine lines, primarily through their ability to stimulate dermal remodeling and promote collagen neogenesis [5]. The development of fractional laser technologies marked a significant advancement in dermatologic interventions. Unlike traditional ablative lasers, fractional systems create microscopic thermal zones, allowing for controlled dermal injury while preserving surrounding tissue [6, 7]. These lasers, such as the 755 nm alexandrite laser with a diffractive lens array and the 1064 nm Nd:YAG laser with a microlens array, create precise subsurface microinjuries through laser-induced optical breakdown. These microinjuries stimulate the skin’s natural repair processes, promoting collagen and elastin production with minimal disruption to the epidermis [5, 8].

Histological analysis indicated increased collagen and elastic fibers in the dermis over time. Compared to traditional laser treatments for facial photorejuvenation, picosecond lasers offer a superior clinical performance and a more comfortable recovery [2]. Recent studies underscore the efficacy of picosecond lasers in reducing wrinkles, improving dyspigmentation, texture, keratosis, facial dyschromia and rhytids without significant changes in erythema [9].

While the clinical efficacy of picosecond lasers is well-documented, the relationship between patient-specific variables—such as skin phototype, baseline skin condition, and treatment parameters—and outcomes remains underexplored. Kirsanova et al. compared the histological effects of low- versus high-fluence picosecond laser treatments, revealing that higher fluence settings achieved more pronounced dermal remodeling but with potentially increased recovery times [2]. This underscores the need for tailored approaches to optimize results while minimizing adverse effects.

In this study, we focused on the use of a 1064 nm Nd:YAG picosecond laser in fractional mode for skin rejuvenation in Asian populations, characterized by a high melanin index and increased susceptibility to post-inflammatory hyperpigmentation (PIH). By analyzing both clinical efficacy and safety, this research aims to elucidate the interplay between epidemiological and clinical variables and treatment outcomes. Through this investigation, we seek to provide insights into optimizing laser protocols to meet the needs of diverse patient populations, ensuring effective and safe rejuvenation with minimal downtime.

## Materials & methods

### Study setting

This study is based on a real-world data analysis, utilizing data from a retrospective chart review from the database of the Department of Dermatology and Skin Aesthetics at University Medical Center HCMC in Ho Chi Minh City, Vietnam. Data analyzed were collected from August 2021 to August 2022. The analysis encompasses patients 18 years of age or older who were diagnosed with aging skin and had an indication to be treated with picosecond Nd:YAG 1064 nm (Discovery Pico Plus, Quanta System S.p.A., Samarate, Italy) at University Medical Center HCMC.

Patients included in the analysis have never treated skin aging with resurfacing procedures such as laser, medium or deep chemical peel within the previous 6 months; intense pulsed light or superficial peel within the previous 1 month; topical therapies with retinoids, alpha-hydroxy acids, salicylic acids within the previous 1 month.

Exclusion Criteria for the study were the following: skin infection, dermatitis or other skin pigmentation disorders on the skin area to be treated, using a self-tanning product within 2 weeks before treatment, using oral medications causing increased photosensitivity, suffering from diseases that increase photosensitivity, patients with underlying skin disease, immunodeficiency, taking hormone replacement therapy, current pregnancy or breastfeeding. Patients who didn’t complete the whole treatment sessions were not included in the statistical analysis.

### Laser settings

Patients received a series of 3 treatment sessions, with a 4-week interval between each session. The whole face was evenly irradiated using the fluence 0.3–0.45 J/cm², pulse duration 450 ps at 10 Hz frequency with the 1064 nm 8 mm deep fractional (8DF) handpiece until the clinical endpoints (slight erythema, mild warmth) were reached. The treatment didn’t combine with an air-cooling system.

### Patient management

Patients received EMLA 5% cream (Eutectic Mixture of Local Anaesthetics, Aspen Pharma UK Limited, UK) for 45 min before treatment, to ensure adequate local anesthesia and enhance patient comfort during the procedure. The patients were not given any topical steroids to help with the erythema or swelling and were ice-packed for 5–10 min before being sent home. Patients were only recommended to apply moisturizers to support barrier recovery evoked by laser treatment and sunscreens for photoprotection.

### Clinical evaluation

In this study, all participants provided signed informed consent prior to enrollment.

Beyond demographic data, photoprotection habits, including daily sun exposure intensity and frequency of sunscreen use, were recorded for each participant. To assess the impact of sun exposure, participants were asked to estimate their average daily time spent in direct sunlight between 9:00 AM and 4:00 PM. This period was chosen to represent the hours of peak solar intensity. Based on these estimates, participants were categorized into one of four groups: no sun exposure, 15–30 min of sun exposure, 30–60 min of sun exposure, or more than 60 min of sun exposure. Additionally, mask-wearing behavior was recorded; given that motorcycles are a prevalent mode of transportation in the study population’s region, the use of sun-protective face masks is widely adopted as a photoprotective behavior for outdoor activities.

Clinical evaluation included the classification of skin aging based on the Glogau Photoaging Classification Scale [10] (see Table 1), categorizing patients into mild, moderate, advanced, or severe photoaging groups.

**Table 1:** Glogau Photoaging classification scale

Group	Classification	Typical age	Description	Skin Characteristics
I	Mild	28–35	No wrinkles	Early photoaging: mild pigmentary change, no keratosis, no or minimal use of foundation, scarce wrinkle
II	Moderate	35–50	Wrinkles in motion	Early to moderate photoaging: early lentigo signs, palpable but not visible keratosis, presence of smile signs, occasional use of foundation
III	Advanced	50–65	Wrinkles at rest	Advanced photoaging: clear sign of dyschromia, visible keratosis, visible capillaries (telangiectasia) always use of foundation
IV	Severe	60–75	Only Wrinkles	Severe photoaging: grayish-yellow skin, history of skin malignancy, wrinkles all over the face, cannot use foundation (cakes and cracks)

Standardized 3D digital imaging using the VISIA^®^ skin analysis system (CANFIELD Imaging Systems) was performed at baseline and at 4, 8, and 12-week follow-up visits. This system objectively measured skin parameters, including texture, wrinkles, porphyrin levels, red areas, pore size, and various spot types. Specifically, the analysis differentiated between superficial spots (brown or red lesions like freckles, acne scars, hyperpigmentation, and vascular lesions), UV spots (epidermal melanin accumulation resulting from sun damage), and brown spots (deeper dermal melanin excesses including hyperpigmentation, freckles, lentigines, and melasma).

For analysis, two distinct metrics obtained from the VISIA camera were utilized: percentiles and absolute scores. VISIA percentiles compare an individual’s skin characteristics to a large database of individuals with similar demographic characteristics, providing a relative ranking. Absolute scores, on the other hand, offer an objective assessment of a specific skin feature, quantifying total size, area, and intensity of the feature [11].

Adverse effects such as erythema, dryness, swelling, and PIH were documented after each session.

Pain levels were recorded using a four-point scale (none, mild, moderate, severe), and subjective improvement was evaluated by patients using a five-point scale ranging from “worse” to “remarkable improvement”. Clinical outcomes were further quantified by reassessing skin aging classification and improvements in VISIA indices across the treatment period.

Through this multifaceted evaluation, the study aims to establish a comprehensive understanding of the clinical efficacy and safety of the fractional 1064 nm Nd:YAG picosecond laser for skin rejuvenation, correlating clinical and epidemiological factors with patient satisfaction and outcomes.

### Statistical analysis

Statistical analysis was performed using Stata computer program version 17.0 (StataCorp. 2023. Stata Statistical Software: Release 17.0. College Station, TX: StataCorp LLC.).

All data were analyzed using descriptive and analytical statistical methods. Descriptive statistics were applied to summarize quantitative variables, expressed as minimum, maximum, mean ± standard deviation (SD), and median with interquartile range (IQR). Categorical variables were presented as percentages.

For analytical statistics, the Wilcoxon signed-rank test was used to assess the mean differences between paired values (e.g., before and after treatment). Relationships between categorical variables were analyzed using the Chi-square test or Fisher’s exact test, as appropriate. A p-value < 0.05 was considered statistically significant.

## Results

**Fig. 1:**
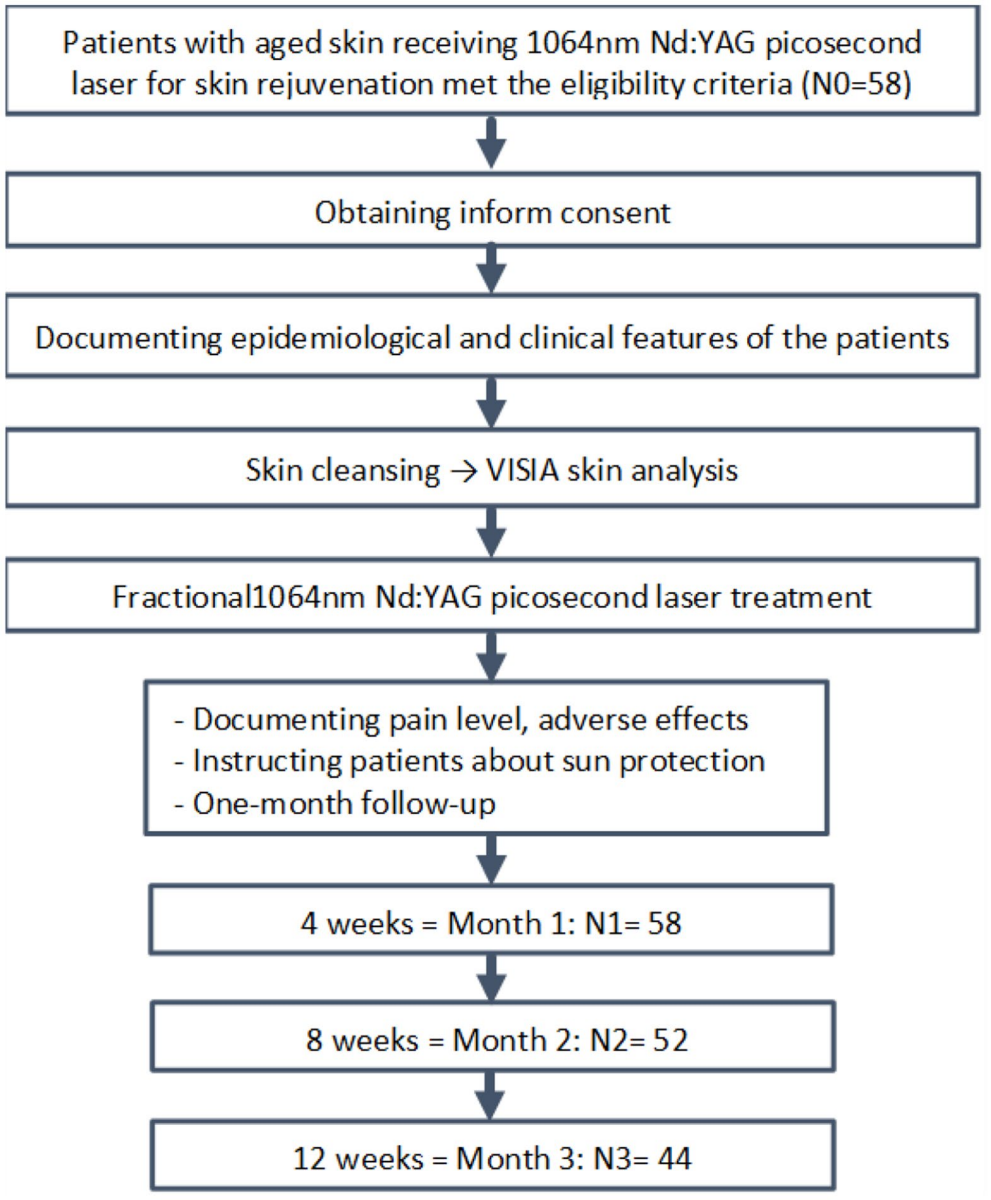
Study enrollment diagram

A total of 58 participants were initially enrolled in this study. Of these, 52 patients received two sessions of laser treatment, and 44 patients completed the study protocol and were included in the study analysis (see Fig. 1 for details).

The mean age of the patients who completed the study protocol was 44.41 years old (IQR: 37–52.2); the youngest patient was 22 years old, the oldest patient was 75 years old (see Table 2).

**Table 2:** Demographic data and skin type distribution

Parameter	Descriptive Statistics
Number of patients	44
Median age	44.41 (37–52.2) years old Min 22 Years old Max 75 Years old
Female/Male	95,45% / 4,55%

### Effectiveness and patient satisfaction

The fractional 1064 nm NdYAG picosecond laser demonstrated high effectiveness and patient satisfaction in treating skin aging. Based on subjective evaluations, 70.45% of participants reported a “moderate improvement”, while 9.09% (4 participants) noted a “remarkable improvement”. A mild improvement was indicated by 8 participants (18.18%), whereas only one subject experienced no improvement (see Table 3).

**Table 3:** Degree of improvement evaluated by subjects

Improvement grade	Patient percentage
None	2,27%
Mild	18,18%
Moderate	70,45%
Remarkable	9,09%

**Fig. 2:**
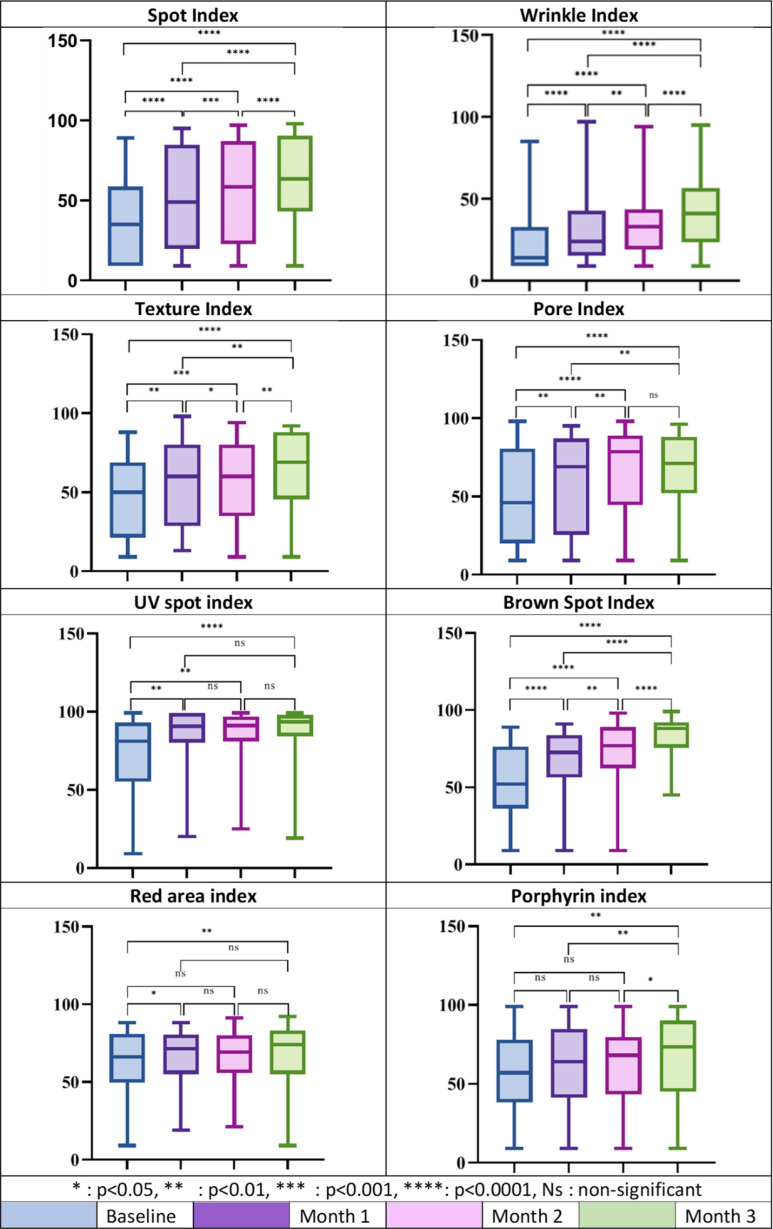
Box plots showing the percentile median and ranges for each VISIA parameter from baseline through Months 1, 2, and 3

The analysis of objective indices using the VISIA skin analysis system demonstrated statistically significant improvements across all facial skin parameters (see Fig. 2). In particular, Spot, Brown Spot and Wrinkle Percentiles showed highly significant improvements at all follow-up points compared to baseline (*p* < 0.0001); similarly, UV spot, Pore and Texture indexes demonstrated marked improvement starting in the first month (*p* < 0.01) and sustained through the third month (*p* < 0.0001). Improvements in Red Area Percentile were evident from the first month of treatment (*p* < 0.05) and became more pronounced in the third month (*p* < 0.01), while a significant improvement in the Porphyrin Index was observed exclusively after the last treatment (*p* < 0.01).

### Adverse effects and pain levels

Adverse effects were minimal and transient. Erythema occurred in 68.18% of patients, while 29.55% experienced swelling. Only 1 participant (2.27%) reported mild dryness (see Table 4).

Pain intensity during the procedure was tolerable, with 61.06% of participants reporting “mild” pain, 34.09% reporting “moderate” pain, and only 2.27% experiencing “severe” pain (see Table 5).

**Table 4:** Percentage of adverse effects

Adverse Effects	Patient percentage
Erythema	68,18%
Swelling	29,55%
Dry Skin	2,27%

**Table 5:** Distribution of pain level

Distribution of Pain	Patient percentage
None	61,06%
Mild	34,09%
Moderate	2,27%
Severe	2,27%

### Relationship between epidemiological and clinical features

The analysis of epidemiological factors, including sun exposure, sunscreen use, and mask-wearing behavior, didn’t reveal important correlations with treatment outcomes. No statistically significant differences were observed analyzing the improvements in skin rejuvenation compared to recorded epidemiological factors (see Table 6).

**Table 6:** Degree of improvement

Category	Value	Total count	Degree of improvement				P-value
			None	Mild	Moderate	Remarkable	
Age	≤ 45	22 (50)	0 (0)	6 (27,27)	15 (63,63)	3 (9,1)	0,32
	> 45	22 (50)	1 (4,54)	2 (9,1)	16 (77,26)	1 (9,1)	
Intense of sun exposure	No	1 (2,27)	0 (0)	0 (0)	1 (100)	0 (0)	0,169
	15–30 min	22 (50)	0 (0)	3 (13,64)	15 (68,18)	4 (18,18)	
	30–60 min	10 (22,73)	1 (10)	4 (40)	5 (50)	0 (0)	
	> 60 min	11 (25)	0 (0)	1 (9,09)	10 (90,91)	0 (0)	
Mask wearing behavior	No	5 (11,36)	0 (0)	1 (20)	3 (60)	1 (20)	0,154
	Often	26 (59,09)	1 (3,84)	2 (7,69)	20 (76,93)	3 (11,54)	
	Always	13 (29,55)	0 (0)	5 (38,46)	8 (61,54)	0 (0)	
Frequency of sunscreen use	No	4 (9,09)	0 (0)	1 (25)	2 (50)	1 (25)	0,055
	Often	33 (75)	1 (3,03)	3 (9,09)	26 (78,79)	3 (9,09)	
	Always	7 (15,91)	0 (0)	4 (57,14)	3 (42,86)	0 (0)	
Glogau photoaging classification scale	Mild	3 (6,82)	0 (0)	1 (33,33)	2 (66,67)	0 (0)	0,75
	Moderate	16 (36,36)	0 (0)	3 (18,75)	11 (68,75)	2 (12,5)	
	Advanced	19 (43,18)	0 (0)	3 (15,79)	14 (73,68)	2 (10,53)	
	Severe	6 (13,64)	1 (16,67)	1 (16,67)	4 (66,67)	0 (0)	

Data are expressed as participant number (percentage). Statistical significance was assessed using Fisher’s exact test to compare the distribution of improvement levels across groups. P-value >  0.05 indicates non-significant differences

Regarding the VISIA parameters analysis, two important findings have been observed: the relationship between history of sun exposure with the therapeutic effectiveness for UV spots (*p* = 0.01) and the relationship between mask wearing habit with the improvement on spots values (*p* = 0.03), both measured after three treatment sessions (see Table 7).

**Table 7:** The improvement on VISIA parameters after 3 treatment sessions

Value	Spots	Wrinkles	Texture	Porphyrin	UV spots	Brown spots	Red areas	Pores
**Age**								
**≤ 45**	29.95± 22.46	22.09± 17.31	22.68± 33.01	19.18± 26.41	18 ± 28.22	30.86± 27.37	10.91± 22.72	5.82 ± 18.91
**> 45**	19.23± 18.50	23.41± 21.99	19 ± 21.03	19.59± 22.37	12 ± 24.97	26.36 ± 20.03	1.95 ± 17.68	11.68 ± 25.77
**P value**	0.11	0.90	0.87	0.61	0.42	0.78	0.30	0.69
**Intense of sun exposure**								
**None**	16	14	20	43	2	63	-2	2
**15–30 min**	25.91± 20.24	27.59± 16.32	26.14± 28.64	16.95± 26.22	17.45± 31.78	31.59± 24.61	5.45± 24.42	8.5 ± 19.97
**30–60 min**	29 ± 25.07	14.1± 24.64	14 ± 32.40	20.1 ± 21.18	27.8 ± 19.10	25.3 ± 25.96	11.2± 12.79	10 ± 26.68
**> 60 min**	18.73 ± 20.26	21.73± 20.15	16.55 ± 20.86	21.45± 24.39	0 ± 10.93	22.55± 19.24	4.82± 19.85	8.73 ± 26.43
**P value**	0.83	0.06	0.63	0.57	**0.01**	0.49	0.45	0.98
**Mask wearing behavior**								
**No**	7 ± 9.75	24.2± 20.05	4.2± 10.47	17 ± 10.98	6 ± 25.96	26.6 ± 7.09	1.4 ± 2.88	-11.8± 17.08
**Often**	29.42± 20.62	24.85 ± 21.96	20.81 ± 30.94	20.88 ± 26.83	15.73± 24.39	31.54± 22.05	7.38± 17.80	11.08± 21.87
**Always**	21.69± 21.94	18± 14.11	27.31± 22.39	17.31± 23.35	17 ± 31.86	23.54± 30.88	6.46± 29.13	12± 22.95
**P value**	**0.03**	0.87	0.14	0.84	0.48	0.40	0.76	0.07
**Frequency of sunscreen use**								
**No**	13.75± 14.93	28.25± 16.26	14.25± 20.73	14.5 ± 9.18	14 ± 23.47	34.25± 10.72	3.25± 6.65	1.75± 5.32
**Often**	26.67± 22.85	21.67± 21.02	24.09± 29.64	20.30± 26.00	15.36± 28.62	29.30± 24.79	8.21± 23.19	9.79 ± 22.40
**Always**	21± 12.70	24.71± 14.96	9.29± 15.53	17.86± 22.86	13.86± 19.76	22.14 ± 25.60	-0.14 ± 9.06	7.86± 30.16
**P value**	0.45	0.59	0.43	0.99	0.89	0.58	0.36	0.55
**Glogau photoaging classification scale**								
**Mild**	30± 38.97	23.67± 22.50	14.33± 56.92	16± 44.24	-0.33± 1.52	40.67± 31.66	37.33± 36.23	-9.67± 11.50
**Moderate**	26.81± 17.67	20.88± 14.48	25.5± 26.65	14.31± 20.53	18.56± 30.12	29.31± 28.21	2.5± 16.14	9.06 22.68
**Advanced**	24.84± 23.62	23.21± 23.77	21.95± 25.93	23.05± 25.92	10.53± 20.85	25.53± 20.38	7.37± 20.91	9.79 ± 22.48
**Severe**	15.17± 10.82	25.83± 20.11	8.17± 17.91	23 ± 20.28	27.33± 36.27	30.5± 21.51	-1.5± 8.78	13.83± 26.68
**P value**	0.66	0.96	0.51	0.47	0.50	0.86	0.14	0.32

Values represent the delta (change) from baseline, calculated using absolute scores, ± SD. P-values < 0.05 indicate significant difference

**Fig. 3:**
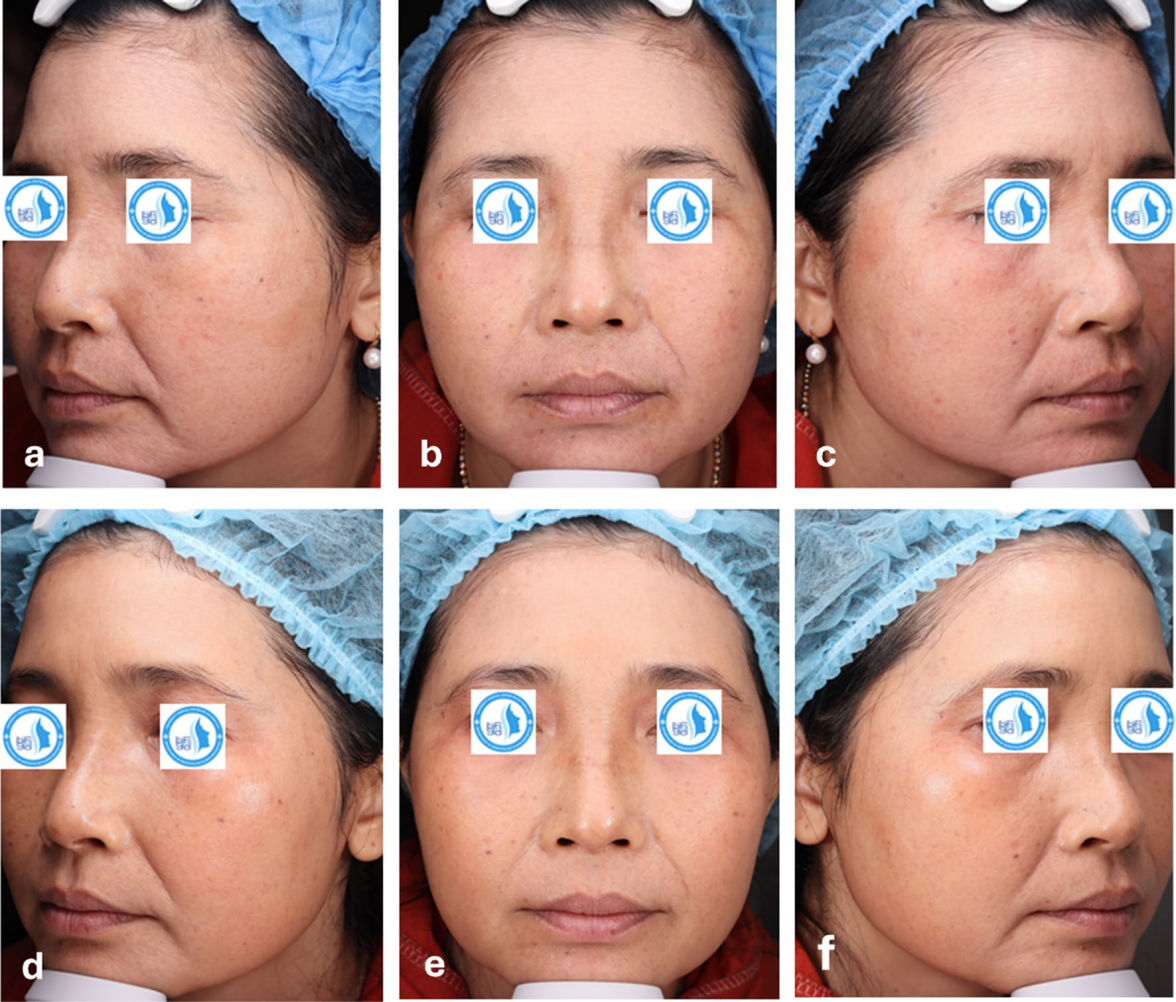
Clinical photographs of a 48-year-old female with Glogau grade III at baseline (**a** left side, **b** front side, **c** right side of the face) and after 3 treatment sessions (**d** left side, **e** front side, **f** right side of the face)

**Fig. 4:**
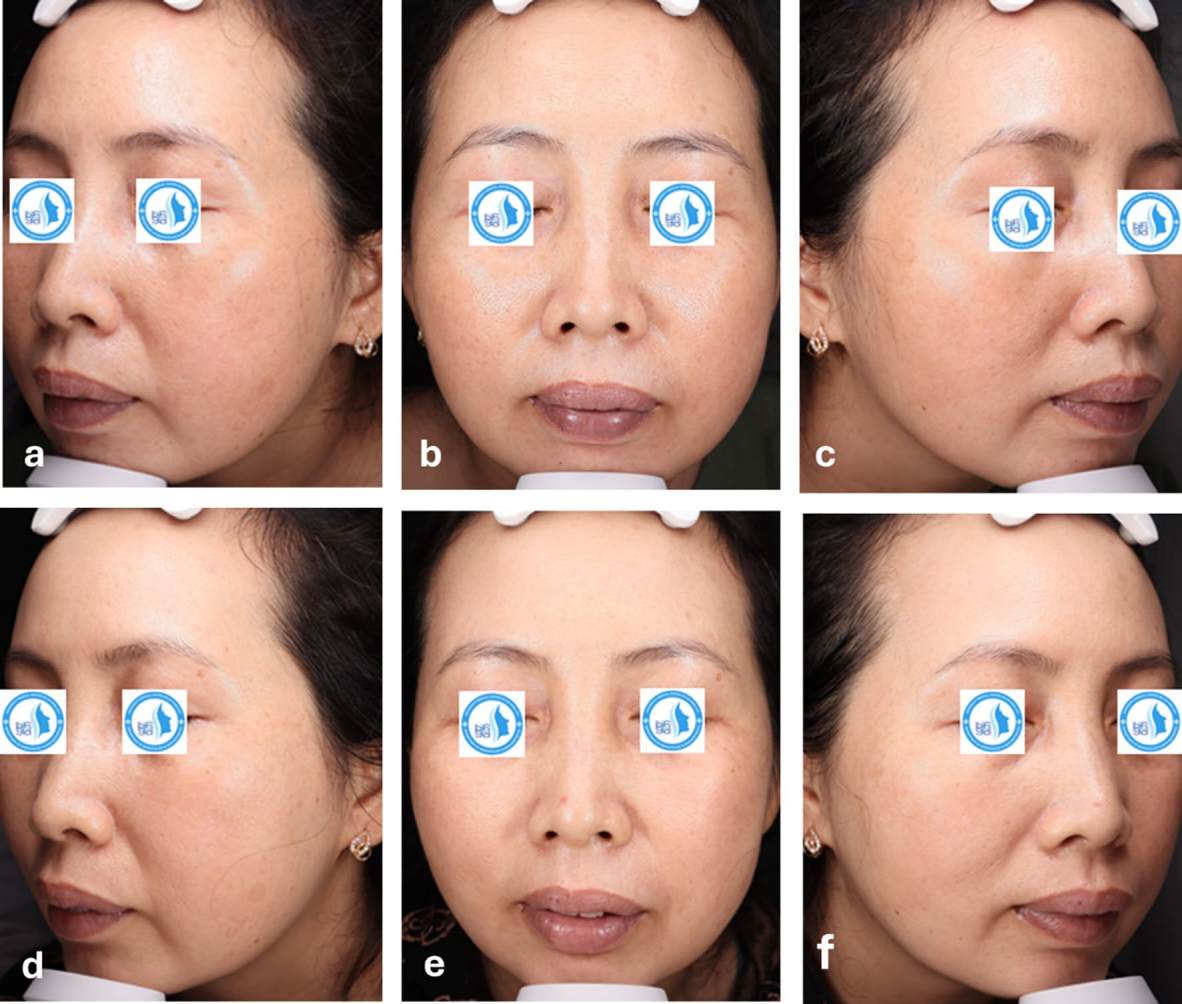
Clinical photographs of a 45-year-old female with Glogau grade III at baseline (**a** left side, **b** front side, **c** right side of the face) and after 3 treatment sessions (**d** left side, **e** front side, **f** right side of the face)

Some clinical cases with a visible improvement after laser treatment are shown in Figs. 3 and 4.

## Discussion

In cosmetic dermatology, the growing demand for facial rejuvenation reflects evolving aesthetic standards and heightened awareness of skin health. This trend is particularly pronounced in Asian cultures, where the emphasis on skin quality and a fair complexion has driven significant growth in the market for whitening products and procedures. The pursuit of flawless, youthful skin aligns with cultural ideals of beauty, often symbolizing purity, vitality, and social status. Facial rejuvenation techniques, including non-invasive and minimally invasive treatments such as lasers, fillers, and chemical peels, have gained popularity due to their efficacy and reduced recovery times. In Asia, the market also reflects a preference for treatments that enhance skin tone uniformity and texture while minimizing pigmentation issues, aligning with consumer demands for quick, visible results and long-term benefits.

The 755 nm picosecond laser has demonstrated considerable efficacy across a range of dermatological conditions, including skin rejuvenation, in Asian population [12, 13]. Its ultrashort pulse duration allows for precise targeting of melanin and other chromophores with minimal thermal damage, making it effective for pigment reduction and textural improvements. However, studies with this technology indicate that relapses may occur over time, and that the risk of PIH is still a notable concern [14, 15].

Laser treatment of Asian skin poses unique challenges due to its higher melanin content compared to Caucasian skin. Melanin, which absorbs light and heat effectively in the 532 to 1064 nm wavelength range, can interfere with laser targeting of dermal lesions and lead to increased absorption of laser energy by the epidermis. Additionally, melanosomes in darker skin are more abundant, larger, and distributed throughout the epidermis, further amplifying the risk of heat-related damage. Compounding this challenge is the slower degradation rate of melanosomes by keratinocytes in dark skin, which prolongs the presence of melanin in treated areas. Overall, this phenomenon can lead to adverse pigmentary effects [16, 17].

The picosecond 1064 Q-switched laser exhibited efficacy and a safety profile in treatment of pigmentary disorders and scars in Asian population [18–20]. This study confirms the effectiveness and safety of the fractional 1064 nm NdYAG picosecond laser for skin rejuvenation in a Vietnamese population. The treatment demonstrated significant improvements across multiple clinical and objective parameters measured by the VISIA skin analysis system, including spots, UV spots, brown spots, wrinkles, texture, pores, and red areas. These findings align with prior studies, reinforcing the versatility and efficacy of picosecond laser technology in addressing various manifestations of photoaging.

The majority of patients (70.45%) reported “remarkable improvement” in subjective evaluations, with high satisfaction levels similar to those reported by Wong et al., who also noted excellent outcomes with picosecond laser treatments for pigmentation and texture improvement [1]. Objective VISIA indices showed statistically significant changes as early as the first month, with continued improvement through the third month, further supporting the laser’s efficacy.

The clinical efficacy of picosecond lasers in mitigating the appearance of wrinkles and unwanted pigmentation has been well established in the literature. Weiss et al., for example, demonstrated significant enhancements in peri-oral and -ocular fine lines after picosecond alexandrite laser treatment [8], while Chen et al. emphasized alexandrite picosecond laser’s capability in treating pigmentation-related concerns [21]. In line with our results, other studies have proven the ability of the 1064 nm picosecond laser in reducing pore size [5] and improving skin texture at a histological level [2]. These findings demonstrate the potential of picosecond laser to address various aspects of skin aging, including both superficial and structural changes. The potential mechanisms for these skin rejuvenation effects have been further explored in a preclinical setting conducted by Lim and colleagues on mouse skin. The study demonstrated that the 1064 nm picosecond laser improved skin texture, increased dermal thickness and blood flow, and stimulated collagen production by disrupting collagen fibers and triggering an inflammatory response [22].

Adverse effects were minimal and transient, with erythema being the most commonly reported side effect (68.18%), resolving quickly without intervention. Pain levels were predominantly mild to moderate, with only 2.27% reporting severe discomfort. These results are consistent with previous studies by Wong et al. and Chayavichitsilp et al., where the safety profile of picosecond lasers was highlighted, emphasizing minimal downtime and manageable discomfort [1, 23].

Interestingly, this study revealed that treatment outcomes were largely unaffected by epidemiological factors such as age, sunscreen use and Glogau photoaging classification scale since no statistically significant difference were observed while analyzing those parameters with respect to the treatment outcome. However, some nuances emerged that merit attention. Patients who reported more than 60 min of daily sun exposure demonstrated a reduced improvement in UV spots (p-value = 0,01), emphasizing the crucial role of sun protection in achieving optimal therapeutic results. Moreover, mask-wearing behavior appeared to influence outcomes, with individuals who consistently wore masks achieving better results for spot reduction compared to those who rarely or never wore them (p-value = 0,03).

These findings highlight the need for tailored patient counseling to optimize outcomes, particularly regarding photoprotection and environmental factors.

This study contributes new insights to the existing body of knowledge by uncovering relationships between specific epidemiological factors and treatment outcomes. Notably, the finding that prolonged sun exposure diminishes the efficacy of UV spot improvement highlights the critical importance of consistent photoprotection. Additionally, the observed positive impact of regular mask-wearing on spot improvement is a novel discovery, which may be linked to reduced environmental exposure or enhanced skin barrier conditions, offering new avenues for optimizing treatment strategies.

### Study limitations

This study is the first to evaluate the efficacy and safety of the fractional 1064 nm Nd:YAG picosecond laser for skin rejuvenation in Vietnam. Additionally, it is the first to explore the relationship between epidemiological factors and clinical outcomes in this context.

Despite the uniqueness of the study, there are several limitations to our study that should be considered when interpreting the results. First, the absence of a control group limits our ability to directly compare the outcomes of picosecond laser treatment with other treatment modalities, such as chemical peels or topical agents. Moreover, the relatively short follow-up period (12 weeks) limits our ability to assess the long-term efficacy and durability of the laser treatment. Skin rejuvenation is a complex process involving collagen remodeling and other biological changes that can continue to evolve over several months or even years. Therefore, it is possible that some of the observed improvements may diminish over time, or conversely, that further improvements could become evident with longer follow-up. Future studies with extended follow-up periods are necessary to evaluate the long-term effects of this laser treatment and determine the optimal maintenance schedule for sustained skin rejuvenation.

## Conclusion

The fractional 1064 nm Nd:YAG picosecond laser is a highly effective and safe option for skin rejuvenation, offering significant improvements in pigmentation, texture, wrinkles, and pores with minimal side effects. These findings reinforce the laser’s role as a versatile tool in dermatologic practice, particularly for Asian populations with higher melanin indices and increased susceptibility to post-inflammatory hyperpigmentation. Counseling on photoprotection and environmental factors, such as mask-wearing, may further optimize outcomes. This study provides a foundation for future research aimed at tailoring treatment protocols to diverse patient populations and exploring the broader applications of picosecond laser technology.

## Data Availability

The data that support the findings of this study are not openly available due to reasons of sensitivity and are available from the corresponding author upon reasonable request.
